# The relationship between flowering time and growth responses to drought in the Arabidopsis Landsberg *erecta* x Antwerp-1 population

**DOI:** 10.3389/fpls.2014.00609

**Published:** 2014-11-11

**Authors:** Inga Schmalenbach, Lei Zhang, Matthieu Reymond, José M. Jiménez-Gómez

**Affiliations:** ^1^Department of Plant Breeding and Genetics, Max Planck Institute for Plant Breeding ResearchCologne, Germany; ^2^Institut Jean-Pierre Bourgin, Institut National de la Recherche AgronomiqueVersailles, France

**Keywords:** mild drought stress, water deficit, flowering time, growth, natural variation, *Arabidopsis thaliana*, epistasis

## Abstract

Limited water availability is one of the most prominent abiotic constraints to plant survival and reproduction. Thus, plants have evolved different strategies to cope with water deficit, including modification of their growth and timing of developmental events such as flowering. In this work, we explore the link between flowering time and growth responses to moderate drought stress in *Arabidopsis thaliana* using natural variation for these traits found in the Landsberg *erecta* x Antwerp-1 recombinant inbred line population. We developed and phenotyped near isogenic lines containing different allelic combinations at three interacting quantitative trait loci (QTL) affecting both flowering time and growth in response to water deficit. We used these lines to confirm additive and epistatic effects of the three QTL and observed a strong association between late flowering and reduced sensitivity to drought. Analyses of growth responses to drought over time revealed that late flowering plants were able to recover their growth in the second half of their vegetative development. In contrast, early flowering, a common drought escape strategy that ensures plant survival under severe water deficit, was associated with strongly impaired plant fitness. The results presented here indicate that late flowering may be advantageous under continuous mild water deficit as it allows stress acclimatization over time.

## Introduction

To ensure their survival and successful reproduction, plants need to respond appropriately to environmental changes. In the context of global climate change, limited water availability is one of the most prominent abiotic constraints to plant survival and productivity in natural environments as well as in crop production systems (Cattivelli et al., 2008). Understanding the molecular and physiological responses to drought in plants can, thus, help us to ensure food production in the future. However, our knowledge about the mechanisms involved in plant's responses to water deficit is hampered by their complexity. Depending on the specific drought scenario and the time of its occurrence during their life cycle, plants combine different strategies involving short-term and long-term responses driven by intricate regulatory networks (reviewed in Chaves et al., 2003). As demonstrated by Skirycz et al. (2011), distinct gene networks are activated depending on the severity of drought stress.

Changes in the rate of growth and/or in flowering time are two common strategies that plants use to cope with drought. Shoot growth inhibition under water deficit helps plants to reduce water loss through transpiration. Recently, Baerenfaller et al. (2012) have demonstrated that plants adapt to an early applied and continuous moderate water deficit by changes in gene expression and by reducing their growth in a constant manner. Interestingly, the transcriptional responses observed differ significantly from modifications of gene expression in the case of a sudden drought stress (Skirycz et al., 2010; Baerenfaller et al., 2012). In terms of flowering time, an early switch from vegetative to reproductive development enables plants to reproduce before the onset of severe water deficit compromises their survival (the so called drought escape strategy; Ludlow, 1989; Sherrard and Maherali, 2006; Franks, 2011). Nevertheless, under a continuous mild water deficit, this strategy can be seen as counterproductive. Early flowering shortens the time available for carbon assimilation during vegetative development and, thus, possibly results in yield reduction. Variation in flowering time has been linked to variation in leaf growth. For instance, in the model species *Arabidopsis thaliana*, delaying the floral transition using short day photoperiods resulted in a reduced final leaf area and leaf expansion rate but an increased duration of leaf expansion (Cookson et al., 2007).

The variation found for growth, flowering time and drought responses among natural accessions in *A. thaliana* is a useful tool to understand plant water relations. Diverse studies have explored variation in physiological parameters in this species, which may play a role in its adaptation to dry environments. In particular, variation for water use efficiency (WUE), a measurement for the trade-off between CO_2_ assimilation and water loss through transpiration, has been studied extensively and used to identify quantitative trait loci (QTL; Mckay et al., 2003; Hausmann et al., 2005; Juenger et al., 2005; Mckay et al., 2008). Furthermore, few genes that underlay drought related traits and, thus, are possibly associated with environmental adaptation have been identified and characterized (Des Marais et al., 2012; Kesari et al., 2012; Lovell et al., 2013). To enable the detailed analysis of plant growth responses to drought, which result from the integration of numerous processes (reviewed in Tardieu et al., 2011), different automated phenotyping systems have been developed. Using one of these systems, Antwerp-1 (An-1) has been identified as one accession with low response of rosette growth to moderate drought stress (Aguirrezabal et al., 2006; Granier et al., 2006). In contrast, Landsberg *erecta* (L*er*) showed a high reduction of rosette growth in response to this drought condition. QTL mapping using a recombinant inbred line (RIL) population derived from a cross between An-1 and L*er* has led to the identification of loci affecting the responses of different growth related parameters to drought (Tisné et al., 2010). Highlighting their complex mode of regulation, drought responses were mainly underlied by epistasis, i.e. QTL x QTL interactions. Using near isogenic lines (NILs) developed for one specific QTL network, Tisné et al. (2010) observed an association between a delay of flowering in response to water deficit and a low response in rosette growth and epidermal cell area and number.

Some works have demonstrated a strong positive correlation between natural variation in WUE and flowering time. This association has been attributed to pleiotropic effects of the flowering time gene *FRIGIDA* (*FRI*) that is epistatic to its downstream target, the floral repressor *FLOWERING LOCUS C* (*FLC*; Johanson et al., 2000; Mckay et al., 2003; Lovell et al., 2013). Plants carrying both functional *FRI* and *FLC* alleles follow a dehydration avoidance strategy characterized by late flowering, slow growth and high WUE. In contrast, non-functional *FRI* alleles result in early flowering, fast growth and low WUE, and, thus, confer a drought escape strategy (Lovell et al., 2013). Indicating an actual relevance of this correlation for adaptation in nature, Stinchcombe et al. (2004) detected an association between the late floral transition of accessions containing a functional *FRI* allele and low January precipitation at their site of origin.

Most studies conducted so far have been focused on the adaptation of plants to severe drought stress endangering their survival and reproduction. Nevertheless, especially in temperate climate zones, moderate but continuous soil water deficit may affect plant productivity. Thus, in the present study, we intend to explore plant strategies to cope with a consistent mild drought stress during vegetative development. In this article we ask whether the timing of flowering, being central for a successful plant reproduction, is associated with growth and fitness responses to mild drought. Making use of previously described natural variation within an *A. thaliana* population (Tisné et al., 2010), we observe a strong association between flowering time and growth in response to water deficit. To further elucidate the genetic basis of this correlation, we develop and characterize a set of near isogenic lines differing in the allelic composition at several interacting QTL affecting both traits analyzed. Monitoring growth responses of the NILs over time leads to the conclusion that late flowering may be advantageous for plant fitness under continuous mild water deficit, as a prolonged vegetative phase may enable plants to recover their growth before flowering.

## Material and methods

### Genetic resources

Several QTL and epistatic interactions affecting growth and flowering time have been previously detected in the Landsberg *erecta* (L*er*) x Antwerp-1 (An-1) RIL population under different water regimes (Tisné et al., 2010). We developed a set of near isogenic lines (NILs) carrying all possible combinations of alleles at three of these QTL (QTL3 x QTL5.1 x QTL5.2) in a homogenous L*er* background. In order to obtain the NILs, we backcrossed RIL102 containing An-1 alleles at all three QTL to L*er*. After two rounds of selfings, we obtained two homozygous NILs carrying a single An-1 introgression at QTL3 or at both QTL5.1 and QTL5.2, respectively. Both NILs were genotyped with 76 Cleaved Amplified Polymorphic Sequence markers distributed across all five chromosomes to confirm the absence of An-1 alleles outside the QTL regions (Supplemental Table 1). Finally, the two lines were crossed to each other and their F_1_ selfed for two generations. In the F_3_ generation, eight homozygous NILs containing the different combinations of QTL alleles were selected (Supplemental Figure 1).

### Drought stress experiment with NILs

Twelve replicates per NIL and treatment were grown in an experiment that mimicked the growth conditions and water regimes of the L*er* x An-1 RIL experiment conducted on the automated phenotyping platform PHENOPSIS (Tisné et al., 2010). Plants were grown in square pots (7 × 7 × 8 cm) containing a standard plant cultivation substrate (Einheitserde Spezial, Typ Mini Tray, Einheitserde- und Humuswerke Gebr. Patzer, Sinntal-Altengronau, Germany) and cultivated under controlled conditions in 12/12 h day/night regimes with temperatures of 22/18°C and an air humidity of 85/75% (day/night). Within each treatment, plants were completely randomized. Before starting the experiment, the initial weight of each pot filled with humid soil was recorded. Subsequently, some pots were removed from the experiment to quantify the initial average soil water content (SWC). To do so, these pots were dried for 3 days at 60°C, weighted afterwards and SWC calculated according to the following formula: SWC = (soil fresh weight—soil dry weight)/soil fresh weight. We assigned this initial SWC to every pot in the experiment. From sowing until the emergence of the first two leaves (growth stage 1.02, according to Boyes et al., 2001), all plants were maintained at 54–60% SWC (72–80% field capacity). From growth stage 1.02 until the end of the vegetative phase (growth stage 6.00), plants grown under well-watered (ww) condition were further maintained at 54–60% SWC, while plants under water deficit (wd) were maintained at 36–42% SWC (48–56% field capacity). The soil water content was adjusted every second day, and, thus, soil dried down over time resulting in the given ranges of SWC in each treatment. To adjust SWC, water was added manually to each pot until the respective target pot weight (corresponding to 60% and 42% SWC in ww and wd, respectively) was reached. This protocol ensured the same water availability for all plants in the respective treatment, independently from possibly different transpiration rates. The experiment was finished for each plant at the time of flowering and for each line when less than three replicates remained without flowering.

From the start of the treatment to the respective day of flowering, we monitored rosette growth by taking photos from above of each plant individually. In these images, we separated the rosette from its background by removing the blue and red color filters and increasing contrast in the green channel in Adobe Photoshop®. Then, images were manually cropped and transformed into binary format using ImageJ. Projected rosette leaf area (RA; mm^2^) was calculated from binary images with the open source ImageJ Plugin Rosette Tracker (De Vylder et al., 2012).

When a plant started flowering, its final projected rosette area, flowering time (FT; in days after sowing) and total leaf number (LN; rosette plus cauline leaves) were recorded. Leaf production rate (LPR; leaves per day) was calculated as the ratio LN/FT. As a proxy for fitness, seed yield (YLD; mg/plant) was measured after complete plant ripening. To determine only the effect of water deficit that occurred during the vegetative phase on YLD, drought stressed plants were grown in ww condition from flowering until complete seed set. The relative response to drought for each parameter was calculated as follows: Response = (trait value in wd—trait value in ww)/trait value in ww.

### *FLC* expression analysis

For analyzing *FLC* expression we used plants grown simultaneously with the plants of the NIL drought experiment described above. All above ground tissues were harvested for each plant 21 days after sowing. Tissue from two plants was pooled for each of three biological replicates. RNA was extracted using Trizol (Ambion® TRIzol® RNA Isolation Reagent, Life Technologies) and transcribed into cDNA using Super Script® II Reverse Transcriptase (Invitrogen). Quantitative RT-PCR was performed on a CFX384 Touch™ Real-Time PCR Detection System (Biorad) using SYBR Green dye (iQ™ SYBR® Green Supermix, Biorad) and the following *FLC* specific primers: F-primer: 5′-CCGAACTCATGTTGAAGCTTGTTGAG-3′, R-primer: 5′-CGGAGATTTGTCCAGCAGGTG-3′. Expression values were determined using the standard curve method and normalized to the expression of *PP2A* (F-primer: 5′-TAACGTGGCCAAAATGATGC-3′, R-primer: 5′- GTTCTCCACAACCGCTTGGT-3′). Normalized expression was averaged for three biological replicates each analyzed in three technical replicates.

### Statistical methods

Data for flowering time (FT), leaf number at flowering (LN) and rosette area (RA) in well watered (ww), and water deficit (wd) conditions for the L*er* x An-1 RIL population was obtained from the PHENOPSIS DB (http://bioweb.supagro.inra.fr/phenopsis/; Fabre et al., 2011). In most cases, four replicates of each RIL were present in each treatment (range from 0 to 4 replicates, mean of 3.73 and median of 4). Values for each individual plant were collected at the time of flowering. To this dataset, we added leaf production rate (LPR) as LN/FT for each individual plant.

Average values and relative responses to water deficit per genotype were calculated to study the correlation between traits and their responses to drought in the L*er* x An-1 RIL population. Mean values per line and trait were calculated by averaging the data from all plants grown both in ww and wd. Relative responses to drought were calculated per RIL and trait as [(mean in wd—mean in ww)/mean in ww]. *P*-values for the correlations between each trait and relative responses to drought were calculated using Pearson's correlation coefficient.

Analysis of phenotypic data from the NILs representing all possible combinations at QTL3, QTL5.1, and QTL5.2 was performed by fitting an ANOVA that included treatment (levels “ww” or “wd”) and genotype at each QTL (levels “L*er*” or “An-1”) together with all possible interactions (phenotype ~ Q3 ^*^ Q5.1 ^*^ Q5.2 ^*^ treatm). The degrees of freedom for FT, LN, LPR, RA, and YLD were 184, 182, 182, 182, and 187 respectively. To study phenotypic differences between individual NILs we defined groups using Tukey's HSD test on the ANOVA mentioned above with a significance threshold of 0.05. Relative responses to drought were calculated per genotype and trait as [(mean in wd—mean in ww)/mean in ww].

For the analysis of *FLC* differences in expression we fitted an ANOVA with the relative expression of *FLC* with respect to *PP2A* (See Material and Methods above) including treatment and genotype at each QTL and with all possible interactions. The ANOVA had 47 degrees of freedoms. Significant classes were obtained using Tukey's HSD test with a significance threshold of 0.05.

## Results

### In the L*er* x An-1 RIL population, flowering time and growth responses to water deficit are positively correlated

**Figure 1:**
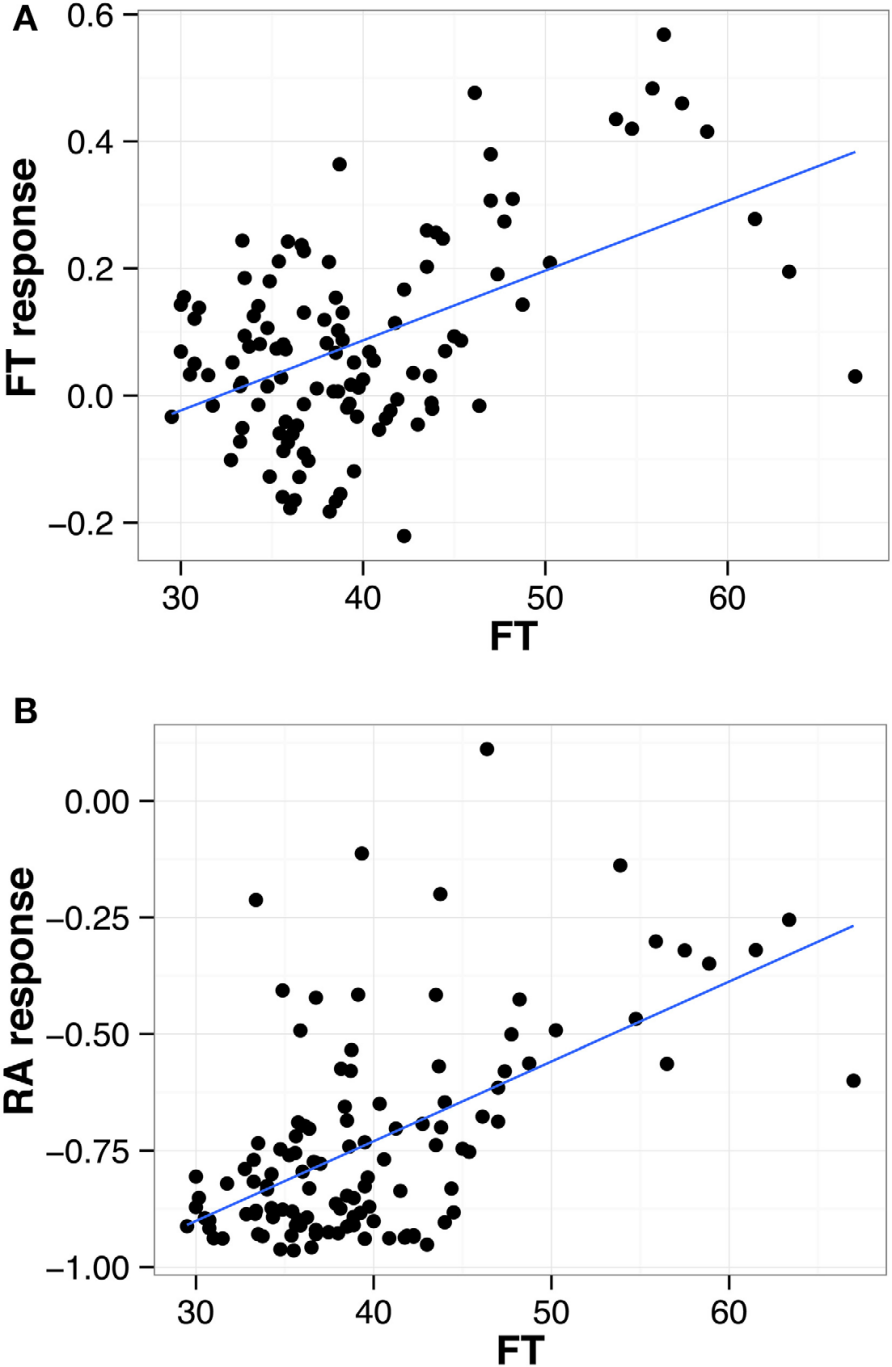
**Correlation between flowering time (FT) and (A) FT in response to water deficit and (B) rosette area (RA) in response to water deficit**. Each dot represents one of the 117 L*er* x An-1 RILs (ww and wd; Tisné et al., 2010). The regression for all points is shown as a solid blue line.

The L*er* x An-1 RIL population (El-Lithy et al., 2006) has been phenotyped previously under two distinct water regimes, namely well-watered control condition (ww) and mild water deficit (wd), using an automated phenotyping platform (Tisné et al., 2010; Fabre et al., 2011). We used flowering time and growth measurements from this experiment to study the association between these traits and their response to drought stress (Fabre et al., 2011, See Material and Methods). We detected highly significant positive correlations between flowering time (FT) and responses of FT and rosette area (RA) to drought (Figure 1, Table 1). Furthermore, these three traits revealed highly significant positive correlations with leaf number (LN) and leaf production rate (LPR, Table 1). Overall, late flowering RILs exhibited a high LPR combined with a delayed floral transition and a low reduction of RA under drought. In contrast, earlier flowering RILs showed a lower LPR and a stronger decrease of RA in response to drought. In addition, early flowering RILs exhibited no FT response or flowered slightly earlier under drought.

**Table 1:** **Pearson's correlation coefficients (*r*) for flowering time, three growth related traits and their relative responses to water deficit in the L*er* x An-1 RILs**.

**Traits**	**LN**	**LPR**	**RA**	**FT response**	**LN response**	**LPR response**	**RA response**
**FT**	0.92^***^	0.83^***^	0.86^***^	0.51^***^	0.62^***^	0.32^***^	0.57^***^
**LN**		0.97^***^	0.94^***^	0.52^***^	0.69^***^	0.40^***^	0.60^***^
**LPR**			0.92^***^	0.51^***^	0.66^***^	0.39^***^	0.58^***^
**RA**				0.64^***^	0.68^***^	0.26^**^	0.51^***^
**FT response**					0.63^***^	−0.14	0.33^***^
**LN response**						0.67^***^	0.75^***^
**LPR response**							0.68^***^

Trait abbreviations are explained in Material and Methods. For calculating correlation coefficients, the means of the performance of each RIL were averaged across the two treatments (ww and wd). The r-values are significant with

** P < 0.01 or

*** *P < 0.001*.

### Confirmation of an epistatic QTL network underlying a complex regulation of flowering time and growth responses to drought using NILs

In the L*er* x An-1 RIL population, a number of QTL have been previously reported for diverse growth related traits such as RA, LN, and duration of the vegetative phase and their responses to drought (Tisné et al., 2010). In multiple cases, QTL for these traits colocalized and presented interactions with each other. For instance, Tisné et al. (2010) detected an epistatic interaction between a QTL on chromosome 3 (at 3.7 cM, in the following named QTL3) and a QTL on chromosome 5 (at 28.4 cM, in the following named QTL5.2) for RA and LN in both water regimes (ww and wd). Furthermore, for both parameters, QTL5.2 interacted with a second QTL on chromosome 5 (at 13.3 cM, in the following named QTL5.1) in wd. In addition, QTL5.1 had an additive effect on RA and LN only in ww condition (Tisné et al., 2010).

In the present study, we developed a set of NILs each carrying one of the eight possible allelic combinations at QTL3, QTL5.1, and QTL5.2 in a homogeneous L*er* background in order to confirm and further characterize the effects of these QTL (Supplemental Figure 1). These lines were grown under two distinct water regimes (ww and wd) mimicking the experimental set up of the RIL experiment described above (for details see Tisné et al., 2010). As in the RIL experiment, the following growth related parameters were quantified at the time of flowering: FT, LN, RA, and LPR. In addition, we measured seed yield (YLD) after complete plant ripening in order to assess fitness differences between the NILs. Finally, we calculated the relative responses to wd using the trait values from both water regimes.

**Figure 2:**
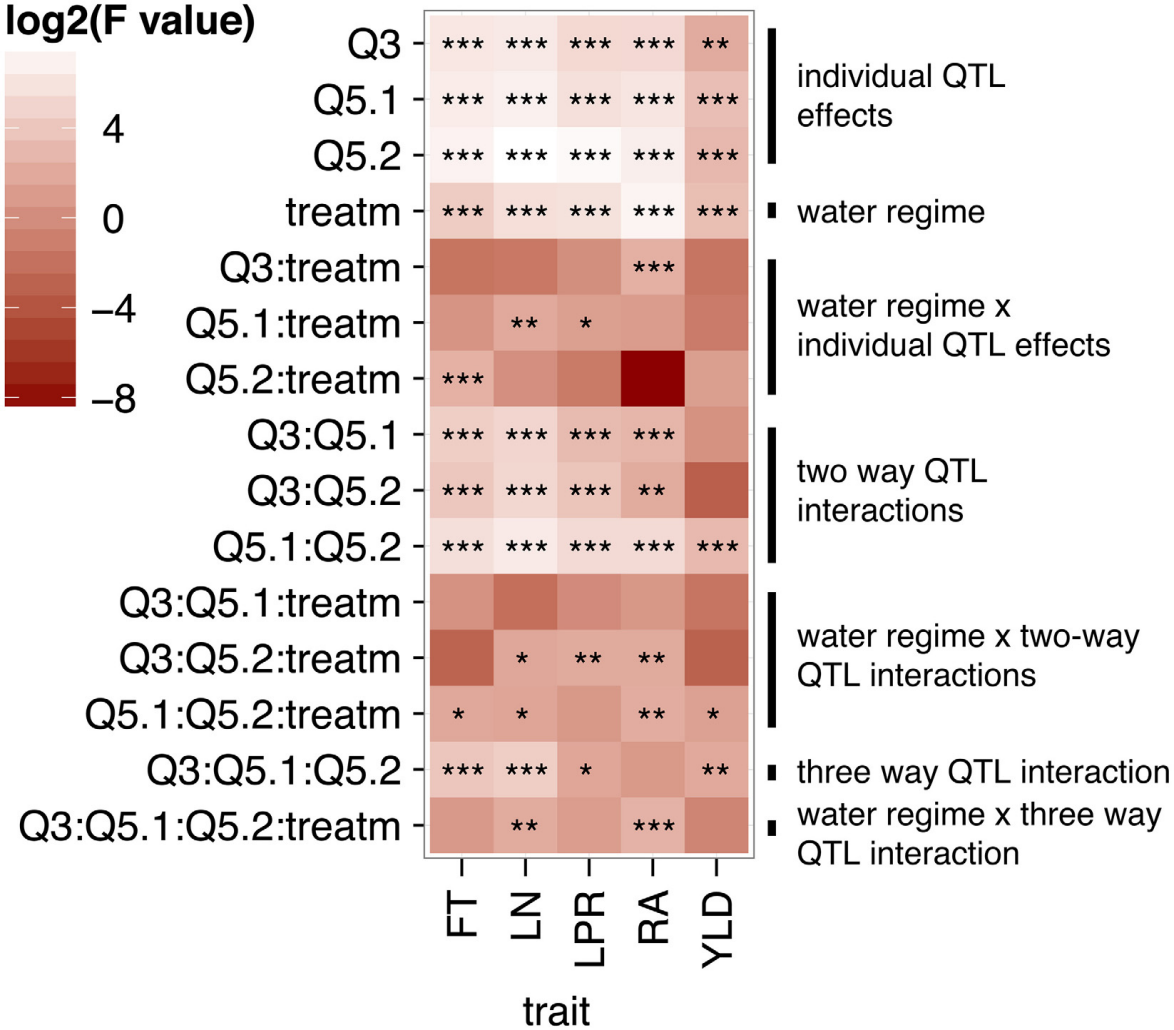
**Heatmap of log2(*F*-values) from an ANOVA conducted for the NIL drought stress experiment**. Genotype at the three interacting QTL (Q.3, Q5.1, and Q5.2) and the water regime (ww and wd) were included as factors in the ANOVA. *F*-values are shown for the effect of individual factors and their interaction. *F*-values that deviate from the null hypothesis are represented with ^*^p < 0.05, ^**^p < 0.01, and ^***^p < 0.001.

An ANOVA using treatment and the genotype at the three QTL as individual factors revealed significant associations between each QTL and all traits analyzed (Figure 2, individual QTL effects). In addition, although the water regime had a strong effect in all traits, most single QTL x treatment interactions only affected individual traits (Figure 2, water regime and water regime x individual QTL effects). Two-way interactions between QTL had significant effects in all traits except YLD, where only the interaction between QTL5.1 and QTL5.2 was significant (Figure 2, two way QTL interactions). Interestingly, interactions involving QTL5.2 exhibited significant effects with treatment for most traits (Figure 2, water regime x two way QTL interactions). Finally, interactions between all three QTL were detected for FT, LN, LPR, and YLD, and between all QTL and environment for LN and RA.

In summary, we confirmed QTL previously detected in the RIL population by Tisné et al. (2010) in lines with a homogenous genetic background. The positive correlation between FT and growth in response to drought observed in the RILs is in part based on additive and epistatic effects of three interacting QTL located on chromosomes 3 and 5.

### Late flowering NILs exhibit a reduced sensitivity to drought

**Figure 3:**
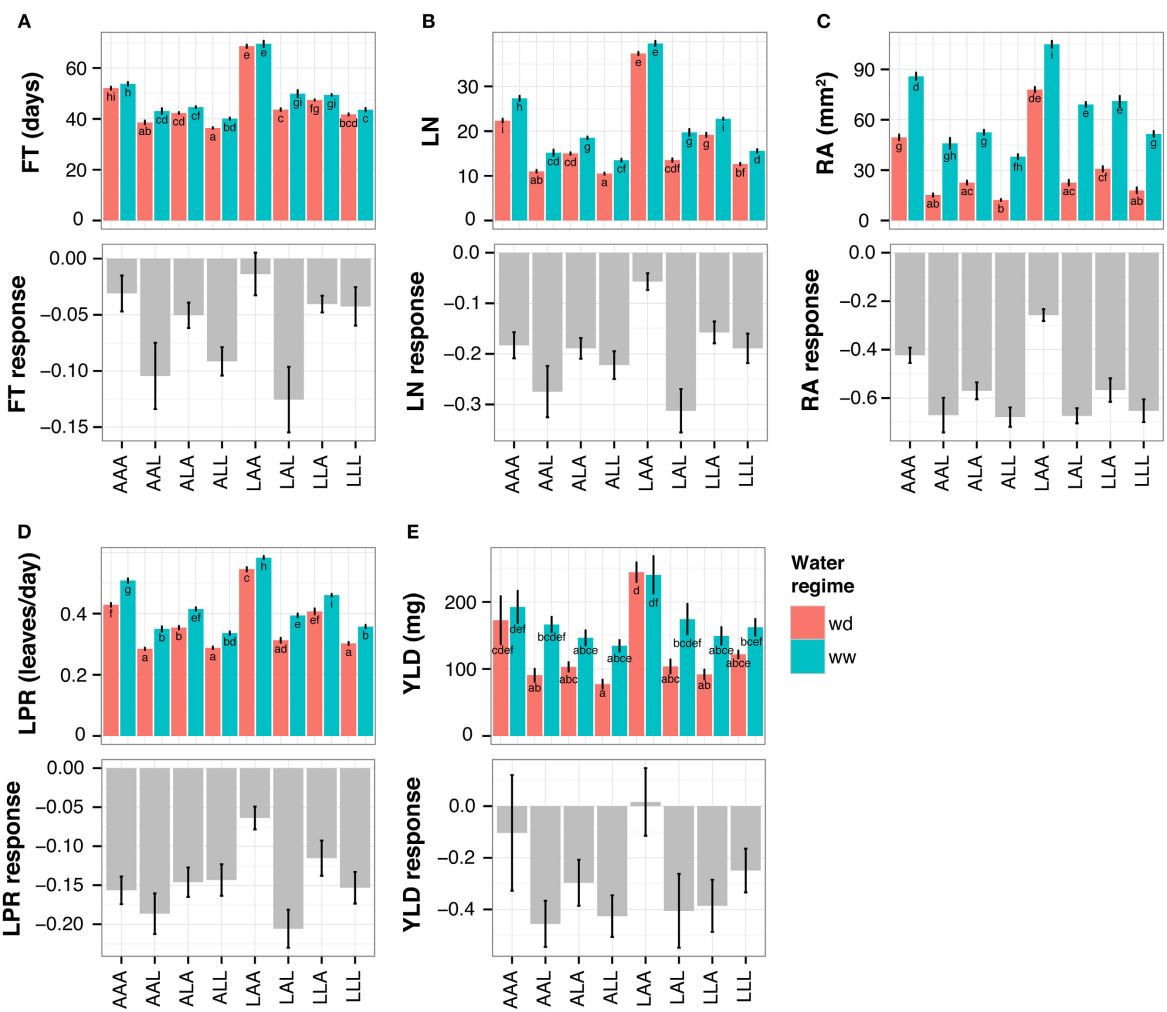
**Trait performances of NILs in both water regimes (upper panel) and in response to water deficit (lower panel, shown as relative response)**. At floral transition the following parameters and their responses to drought were quantified: **(A)** flowering time (FT; in days after sowing), **(B)** total leaf number (LN), **(C)** projected rosette area (RA) and **(D)** leaf production rate (LPR). Furthermore, after seed ripening **(E)** seed yield (YLD) and its response were quantified. The NILs are named according to their allelic combination at QTL3, QTL5.1, and QTL5.2, where the An-1 and Ler alleles are abbreviated by “A” and “L,” respectively. Average trait values for 8–12 plants per NIL and treatment (mean of 11.5, median of 12). Wd, water deficit; ww, well-watered condition. Error bars indicate the standard error of the mean. Distinct letters indicate significant differences calculated with a Tukey's HSD test from an ANOVA with treatment and genotype at each QTL as factors, including all possible interactions.

The small genetic variation and large phenotypic differences present among the NILs is an ideal tool to study the relationship between flowering time and growth responses to drought. As expected from the ANOVA described above, the lines displayed significant variation for these traits in both water regimes as well as for their response to drought (Figure 3). With some exceptions, all lines showed a reduction of all parameters in wd condition, especially in RA (Figure 3C).

The line carrying L*er* alleles at QTL3 and An-1 alleles at both QTL5.1 and QTL5.2 (in the following called NIL LAA, for L*er*:An-1:An-1 at QTL3:QTL5.1:QTL5.2) flowered the latest and had the highest RA, LN, LPR, and YLD in both conditions (Figure 3). In agreement with the correlations observed in the RILs (Figure 1, Table 1), these phenotypes were associated with the lowest growth response to drought, i.e. the lowest reduction of RA in wd (Figure 3C). Moreover, line LAA did not change its LN in response to drought, whereas all other NILs had significantly less leaves in wd than in control condition (Figure 3B). The low growth response of NIL LAA was associated with maintained fitness under drought. As shown in Figure 3E, line LAA had the highest YLD under both conditions and exhibited no response to drought for this parameter. In contrast, YLD was considerably reduced in wd in all other lines except AAA that flowered the second latest and exhibited the second lowest growth reduction among all lines.

In agreement with the correlations detected in the RILs, late flowering was associated with low growth responses to drought in the NILs. As, furthermore, these phenotypes were associated with stable fitness under drought, late flowering seem to be favorable under the mild drought condition applied here.

### Late flowering allows drought acclimatization during the second half of vegetative development

The phenotypic differences at the time of flowering among RILs and NILs described above reflect the culmination of a drought response strategy that has occurred during the vegetative phase of the plants' life cycle. In order to describe the different strategies to cope with water deficit in detail, we monitored plant growth over time in the NILs. We quantified RA from images of plants taken approximately every 2 days in ww and wd conditions and calculated the relative RA response for each time point. Sample images from the earliest and latest flowering NIL, respectively, are shown in Supplemental Figure 2.

**Figure 4:**
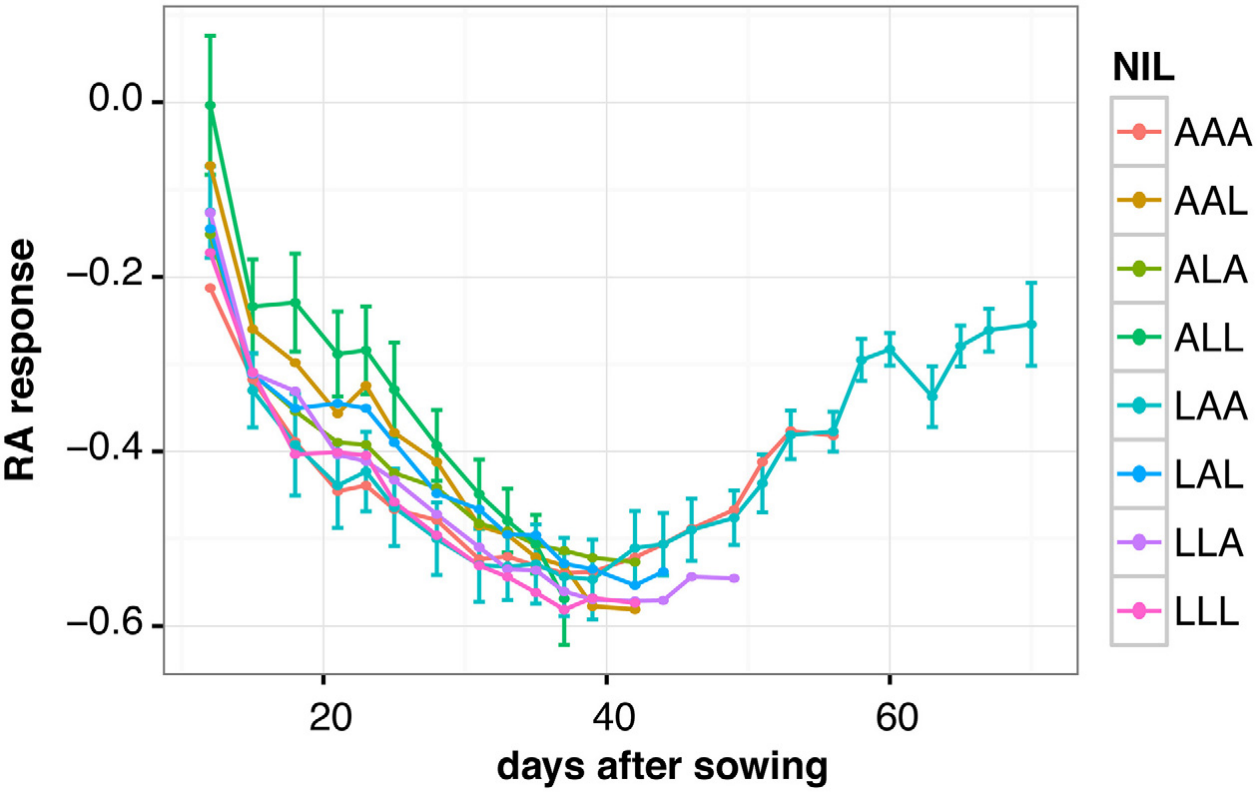
**Relative response of rosette area (RA) to water deficit in NILs over time**. The NILs are named according to their allelic combination at QTL3, QTL5.1, and QTL5.2, where the An-1 and L*er* alleles are abbreviated by “A” and “L,” respectively. Average responses for 3 to 12 plants per line and time point are shown. Error bars indicate the standard error of the mean. For the sake of clarity, error bars are only shown for the two NILs flowering the earliest (ALL) and the latest (LAA), respectively.

We observed two different phases in the drought responses of the NILs. For approximately the first 40 days of the experiment, the differences between control and drought grown plants increased steadily for all NILs, and no significant variation in this pattern was detected between the lines (Figure 4). All lines displayed a progressive reduction of rosette growth under wd. After this period, the early lines reached the end of their vegetative phase, i.e., they started flowering (e.g., NILs ALL and AAL). Interestingly, the late flowering NILs LAA and AAA were able to recover during the second half of their vegetative phase, as indicated by a continuous decrease in their RA response over time (Figure 4). During this period, the late lines grew faster in wd than in ww control condition, enabling them to compensate for previous growth deficits (Supplemental Figure 3). As NIL LAA flowered the latest, it had most time to recover, resulting in the lowest reduction of RA in response to wd at its transition to the reproductive phase (Figure 4, Figure 3C).

In conclusion, analysis of dynamic growth responses revealed an advantage of a late floral transition under continuous moderate drought stress as it allows plants to recover from water deficit over time before setting seeds.

## Discussion

### An epistatic QTL network controls variation in flowering time, growth and their responses to water deficit in the L*er* x An-1 population

In order to study the relationship between FT and growth in response to a continuous mild drought stress, we reanalyzed data of the L*er* x An-1 RIL population grown under two water regimes (Tisné et al., 2010). We detected highly significant positive correlations between FT and several growth related parameters in general, i.e. averaged across the two water conditions, and in response to wd (Figure 1, Table 1). Most notably, late flowering RILs delayed their floral transition in response to wd, which was associated with low growth responses to drought. Making use of NILs, we demonstrate here that these correlations are, at least partially, controlled by an epistatic QTL network involving three QTL. In this framework of interacting loci, allelic combinations that resulted in late flowering conferred less reduced growth and stable fitness under drought, and, thus, can been seen as favorable under mild water deficit conditions (Figure 3). This association between flowering time and growth in response to drought is in agreement with the positive correlation detected in the RIL population. However, one has to be aware that in the NIL experiment, growth has been quantified as projected rosette leaf area (RA). Using this method, an overlap of leaves that appear later in development with older leaves (see Supplemental Figure 2, time points 56 and 70 d.a.s) might bias the growth measurements, and, thus, the quantification of the RA response to wd. Nevertheless, two observations support that the differences in the response to drought detected between late and early NILs are real. First, similar results as ours were obtained in the RIL experiment where RA was determined from scans of individual leaves (Tisné et al., 2010). Second, the reduced response to drought observed for the growth of late NILs was also observed for their yield, a trait that has been measured independently from the images.

Interestingly, the late flowering NILs did not delay FT in response to drought as detected for late flowering RILs (Figure 1), but displayed no change in FT under drought, whereas early flowering lines accelerated floral transition slightly (Figure 3A). A reason for this discrepancy between the NIL and the RIL experiment might be that plants in the NIL experiment were unintentionally subjected to a less severe drought stress than the RILs resulting in an overall smaller response of FT. Although we tried to mimic the experimental conditions of the RIL experiment, unavoidable differences due to for instance micro-meteorological variation might have occurred. Nevertheless, in both experiments, the late flowering lines reduced their growth less than the early flowering lines (Figures 1B, 3A,C).

Our results raise the question of whether the correlations observed are specific for the genetic material studied here or if they are a general phenomenon in *A. thaliana*. Aguirrezabal et al. (2006) analyzed growth responses to soil water deficit of 25 natural accessions and did not detect any correlations between leaf number (which is a common measurement of FT) and responses to drought in leaf number or RA. Furthermore, An-1, which is early flowering, was shown to maintain its growth better under drought than other accessions that are known to flower significantly later (Shindo et al., 2005; Granier et al., 2006). These results suggest the correlations we detected here are rather specific. More comprehensive studies with for instance flowering time mutants or other segregating populations would be needed to corroborate a general association between FT and growth responses to drought.

In the present study, we confirm and further characterize a complex QTL network with pleiotropic effects on two major plant traits and their responses to moderate drought stress. Multiple QTL studies in *A. thaliana* and other species have demonstrated that the analysis of epistasis (QTL × QTL interactions) is essential for describing the architecture of quantitative traits (reviewed in Mackay, 2014). Although epistasis between two QTL has been detected frequently, analyses of higher dimensional interactions like the one reported here are so far rare.

### *FLC* and *HUA2* are candidate genes for QTL5.1 and 5.2, respectively

As described previously, the two QTL on chromosome 5 are close to genes known to affect flowering time (Tisné et al., 2010). These are *FLC* (close to QTL5.1) and the putative transcription factor *HUA2* (close to QTL5.2) that up regulates expression of *FLC* (Doyle et al., 2005). Both genes are reasonable candidates for the detected QTL as both of them exhibit functional polymorphisms for the two parental accessions. Whereas L*er* carries a weak *FLC* allele whose expression is inhibited by an intronic transposon (Liu et al., 2004), An-1 contains a stronger allele (El-Lithy et al., 2006). Thus, we hypothesize that the variation in flowering time we observed among NILs is associated with differences in *FLC* expression. Indeed, the two NILs flowering the latest, LAA and AAA, had the highest *FLC* expression level in both water conditions. In contrast, early flowering lines displayed low expression or no expression at all (Supplemental Figure 4). Furthermore, L*er* contains a mutation at *HUA2* (*hua2.5*) resulting in a premature stop codon, and, thus, impairing the up regulation of *FLC* (Doyle et al., 2005). We confirmed this mutation in L*er* and its absence in An-1 (data not shown). The allelic nature of *FLC* and *HUA2* in the two parental accessions is in agreement with the epistatic effects observed here. The presence of strong An-1 alleles at both *FLC* and *HUA2* (NILs LAA and AAA) is associated with high *FLC* expression levels and a late floral transition. Thus, we hypothesize that the QTL network studied here is based on a *FLC* dependent regulation involving different *FLC* regulators, such as *HUA2* and an unknown gene at QTL3. Supporting this hypothesis, Tisné et al. (2010) described a similar QTL network including QTL5.1, QTL5.2 and a QTL on chromosome 4 that also regulates FT and growth in response to drought. Here, as well, late flowering lines showed a low growth response to wd, probably based on the up-regulation of *FLC* through its main regulator *FRI* (Johanson et al., 2000), a possible candidate gene for the QTL on chromosome 4 (Tisné et al., 2010).

Several studies have linked *FLC* and plant-water relations. Different models including *FLC* as one regulator have been proposed for the regulation of drought responsive flowering (Riboni et al., 2013; Xu et al., 2014). Furthermore, a *FLC* dependent pleiotropic effect of *FRI* on flowering time, growth rate and WUE has been reported as described below in more detail (Mckay et al., 2003; Lovell et al., 2013).

Nevertheless, further analyses, such as QTL fine-mapping, cloning, and complementation, would be required to confirm the candidate genes proposed here.

### Late flowering enables drought acclimatization over time

In our lines, a late floral transition was strongly correlated with a reduced sensitivity to drought, and, thus, we assumed that late flowering plants follow a specific strategy to cope with soil water deficit. To test this, we quantified dynamic growth responses of the NILs. Although in the first half of the experiment, we could not detect any significant differences between early and late flowering genotypes, we observed a recovery of late flowering lines over time resulting in the lowest reduction of RA at floral transition. These results lead to the assumption that it is crucial for a plant at which developmental stage it is hit by drought stress. In our study, the drought treatment started for all genotypes at the same time (See Material and Methods) neglecting their different developmental stage. Thus, for early flowering plants, the wd occurred late during their vegetative phase when they already had initiated flowering. These plants had probably no time to acclimatize to the stress due to their early transition to the reproductive phase. As a consequence, growth and fitness were strongly impaired under wd and flowering slightly accelerated. In contrast, late flowering lines seemed to be able to acclimatize to the stress, possibly due to diverse physiological adjustments, as it occurred early during their life cycle. In addition, as the length of the vegetative phase was the same for late flowering lines in ww and in wd, the drought stressed plants were able to compensate previous growth deficits better than early flowering lines in wd. Based on the observations of Tisné et al. (2010) in the same population, we speculate that the late flowering NILs are able to maintain both epidermal cell area and cell number under wd better than all other lines, resulting in less reduced rosette size at floral transition. Furthermore, Juenger et al. (2005) observed a positive correlation between flowering time and WUE in the L*er* x Cape Verde Island (Cvi) RIL population, where late flowering lines exhibited higher WUE. Similarly, Lovell et al. (2013) established a link between WUE and growth rate through variation in *FRI*. Functional *FRI* alleles confer a dehydration avoidance strategy in which plants are late flowering, have a higher WUE and decreased growth rate. This effect of *FRI* was only observed in the presence of a functional *FLC* allele (Mckay et al., 2003; Lovell et al., 2013). Thus, we may hypothesize that our late flowering NILs carrying functional *FLC* alleles and showing increased *FLC* expression (Figure 4) may exhibit a higher WUE than the early flowering genotypes. In addition, we may assume that the overall physiological state of our NILs is affected by allelic variation at QTL3. Juenger et al. (2005) detected pleiotropic effects of a QTL colocalizing with our QTL3 on flowering time and WUE in the L*er* x Cvi RILs. Analysis of a NIL revealed that presence of the L*er* allele at this QTL does not only result in increased flowering time and WUE, but also in lower stomatal conductance and higher transpiration efficiency. Furthermore, the L*er* allele was associated with a decreased water loss rate measured from whole rosettes over time (Juenger et al., 2005). Whether these characteristics are advantageous under water deficit is not clear. However, to get insight into the mechanisms underlying the drought acclimatization strategy of our late flowering lines, comprehensive analyses, such as physiological studies, genome wide gene expression (Des Marais et al., 2012) or metabolite studies (reviewed in Verslues and Juenger, 2011), would be required.

In summary, our quantification of growth responses over time revealed two distinct phases of drought responses. The first phase was characterized by a strong reduction of growth due to water deficit in all lines, regardless their flowering time. Whereas most of the lines flowered after this phase, late flowering lines exhibited a second phase of growth recovery from drought. Only their late floral transition enabled them to compensate growth deficits resulting from wd through increased growth rates. In contrast, early flowering genotypes followed a drought escape strategy, which is known as a common mechanism to enable plant survival and reproduction before lethal drought conditions occur (Ludlow, 1989). Under the continuous moderate drought stress we applied here that was characterized by short cycles of drying out and re-watering (See Material and Methods), such a drought escape strategy resulted in strongly impaired growth and reduced fitness. In contrast, a late floral transition allowed plants to acclimatize to wd over time, and thus, proved to be advantageous for plant fitness under moderate drought stress.

### Conflict of interest statement

The authors declare that the research was conducted in the absence of any commercial or financial relationships that could be construed as a potential conflict of interest.
