# Supplementary figures and images for: The relationship between flowering time and growth responses to drought in the Arabidopsis Landsberg erecta x Antwerp-1 population
Source: Front Plant Sci. 2014 Nov 11;5:609. doi: 10.3389/fpls.2014.00609 (PMC4227481; doi:10.3389/fpls.2014.00609)

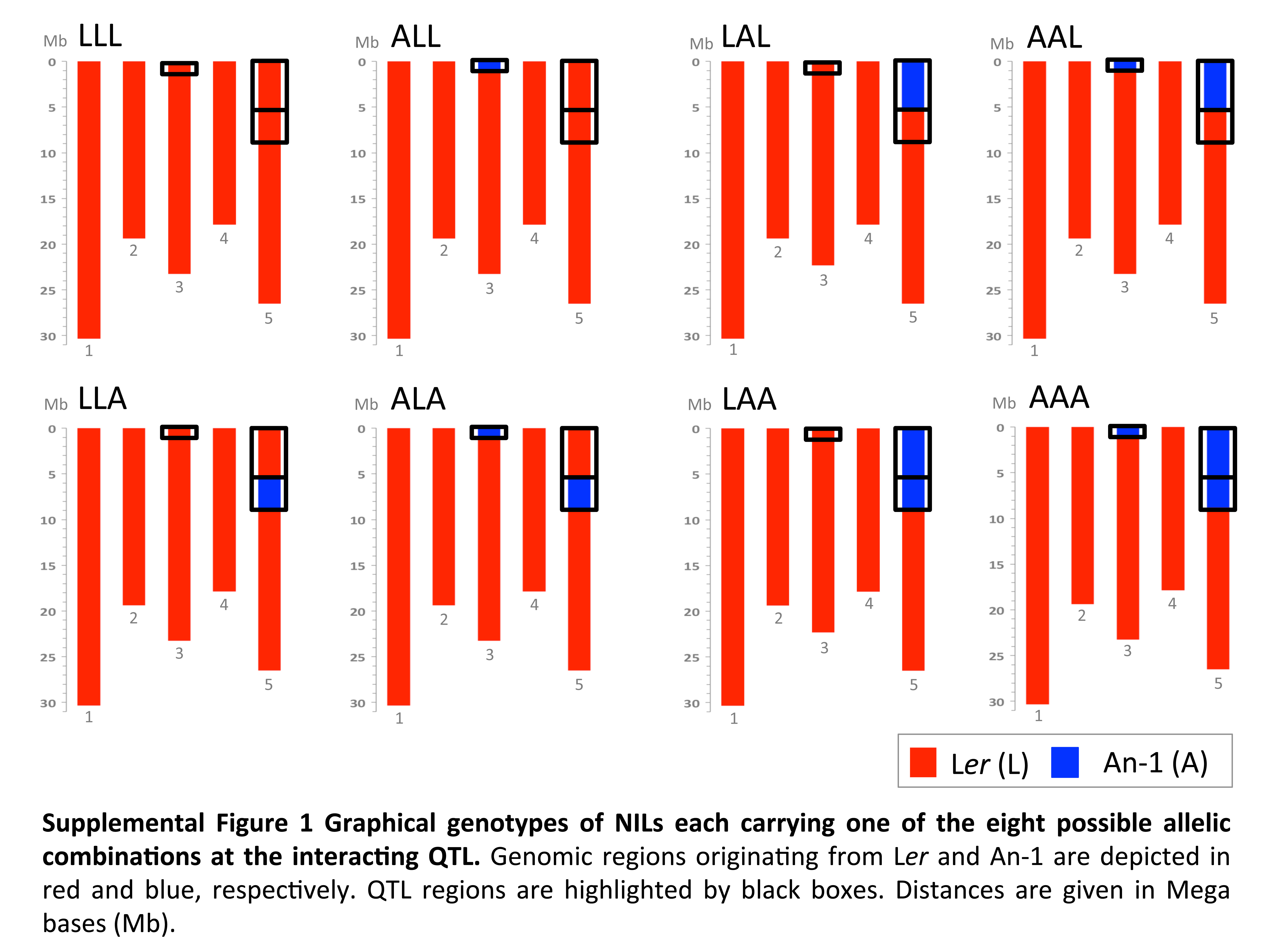

Supplement: Supplementary file 2 [file Image1.JPEG]

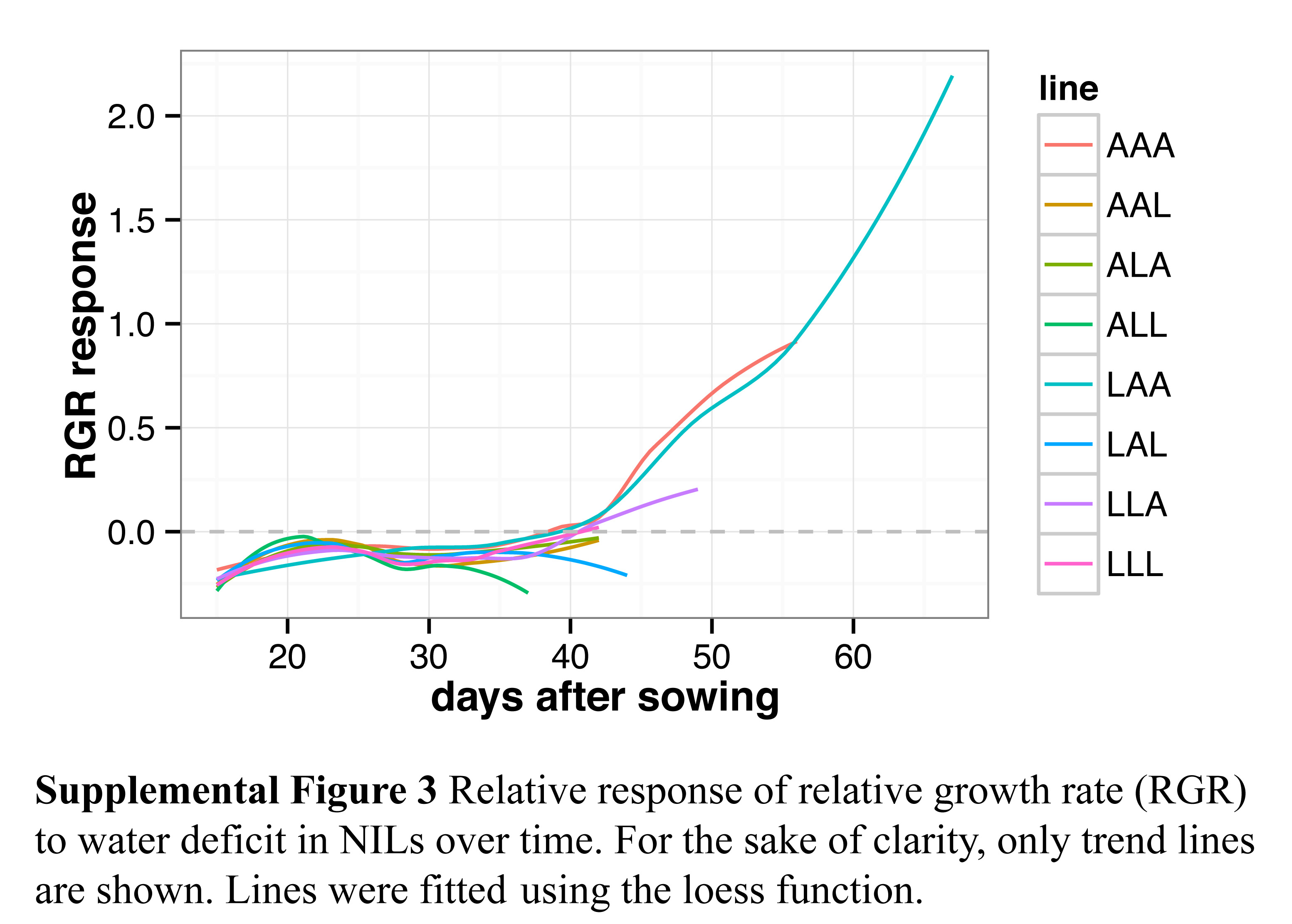

Supplement: Supplementary file 4 [file Image3.JPEG]
