# Supplementary material for: The relationship between flowering time and growth responses to drought in the Arabidopsis Landsberg erecta x Antwerp-1 population
Source: Front Plant Sci. 2014 Nov 11;5:609. doi: 10.3389/fpls.2014.00609 (PMC4227481; doi:10.3389/fpls.2014.00609)
Supplement: Supplementary file 5 [file Image4.PDF]

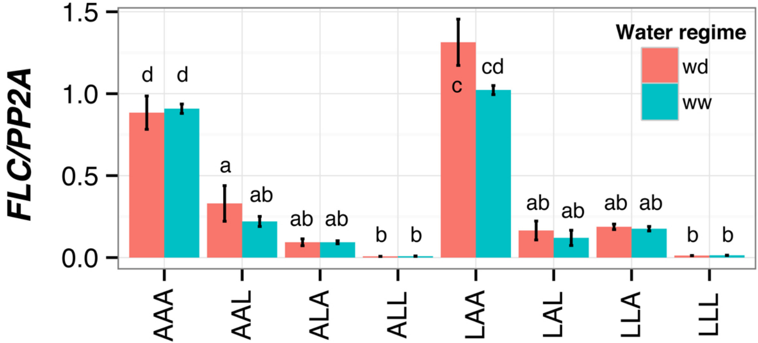

Supplemental Figure 4 Relative *FLC* expression levels in the NILs in well-watered control condition (ww) and water deficit (wd). Plants were grown in a growth chamber at 12h day length and sampled three weeks after sowing. FLC expression was quantified using quantitative RT-PCR and normalized to expression of *PP2A*. *FLC* levels are shown as average values for three biological replicates per line and treatment. Error bars represent the standard error of the mean. Distinct letters indicate significant differences calculated with a Tukey's HSD test from an ANOVA with treatment and genotype in each QTL as factors, including all possible interations.
